# Comparison of Immediate and 2-Year Outcomes between Excimer Laser-Assisted Angioplasty with Spot Stent and Primary Stenting in Intermediate to Long Femoropopliteal Disease

**DOI:** 10.1155/2013/247102

**Published:** 2013-12-05

**Authors:** Tien-Yu Wu, Hsin-Hua Chou, Shang-Hung Chang, Yueh-Ju Tsai, Chien-An Hsieh, Shih-Tsung Cheng, Kuan-Hung Yeh, Hern-Jia Chang, Yu-Lin Ko, Hsuan-Li Huang

**Affiliations:** ^1^Section of Cardiology, Department of Medicine, Taipei Tzu Chi Hospital, The Buddhist Tzu Chi Medical Foundation, 289 Jiang Kuo Road, Xindian District, New Taipei City 231, Taiwan; ^2^Section of Cardiology, Department of Medicine, Chang Gung Memorial Hospital, 5 Fusing Street, Gueishan Township, Taoyuan County 333, Taiwan; ^3^Department of Ophthalmology, Chang Gung Memorial Hospital, 5 Fusing Street, Gueishan Township, Taoyuan County 333, Taiwan

## Abstract

*Background*. To compare the clinical outcomes between excimer laser-assisted angioplasty (ELA) with spot stent (group A) and primary stenting (group B) in intermediate to long femoropopliteal disease. *Methods*. Outcomes of 105 patients totaling 119 legs treated with two different strategies were analyzed retrospectively in a prospectively maintained database. *Results*. Baseline characteristics were similar in both groups. Better angiographic results and lesser increase of serum C-reactive protein levels (0.60 ± 0.72 versus 2.98 ± 0.97 mg/dL, *P* < 0.001) after the intervention were obtained in Group B. Group A had inferior 1-year outcomes due to higher rate of binary restenosis (67% versus 32%, *P* = 0.001) and lower rate of primary patency (40% versus 58%, *P* = 0.039). Rates of amputation-free survival, target vessel revascularization, assisted primary patency, and stent fracture at 24 months were similar in both groups (80% versus 82%, *P* = 0.979, 65% versus 45%, *P* = 0.11, 78% versus 80%, *P* = 0.75 and 6.3% versus 6.8%, *P* = 0.71, resp.). *Conclusion*. Greater vascular inflammation after ELA with spot stent resulted in earlier restenosis and inferior 1-year clinical outcomes than primary stenting. This benefit was lost in the primary stenting group at 2 years due to late catch-up restenosis. Active surveillance with prompt intervention was required to maintain the vessel patency.

## 1. Introduction

The best treatment strategy of endovascular intervention (EVI) for intermediate to long femoropopliteal disease remains uncertain. Restenosis rates after percutaneous transluminal angioplasty (PTA) vary from 40 to 60% at 1 year [1, 2], with up to 70% failure at 1 year after PTA of lesions >10 cm in extensive superficial femoral artery (SFA) disease [2].

Recent studies have demonstrated superior short- and midterm patency provided by nitinol self-expanding stents when compared to PTA alone in treating lesions with a length of up to 15 cm in the SFA and proximal popliteal artery [3–6]. Femoropopliteal artery in-stent restenosis (ISR) remains problematic, occurring in 19–37% of cases after 1 year [5–7]. Besides, stent fracture is another important concern, especially for longer lesions. The incidence of stent fracture has been shown to increase with increasing lesion length and major stent fractures have been associated with restenosis or reocclusion [8]. The excimer laser application has been studied in long SFA occlusions as well as below-the-knee lesions in patients with critical limb ischemia (CLI). Laser debulking followed by adjunctive balloon angioplasty has been shown to provide better immediate angiographic results and can also reduce the need for stenting, especially in long femoropopliteal occlusions. Besides, this debulking may change the practice from an “extensive stenting” to a “focal spot stenting” [9]. There is a paucity of information in the literature regarding the focal spot stent versus primary stenting in the femoropopliteal disease. This study was designed to compare the clinical outcomes between ELA with spot stent and primary stenting in the treatment of intermediate to long femoropopliteal disease.

## 2. Methods

From March 2008 to December 2012, a total of 105 patients with 119 legs having intermediate to long femoropopliteal lesions were enrolled in this study. All patients were informed of the risks and benefits of participating in the study and gave written consent to participate before enrollment. Patients eligible for inclusion in this study were more than 18 years old; had symptoms of intermittent claudication or CLI (Rutherford-Becker categories from 3 to 6); were candidates for endovascular treatment; had de novo stenotic, occlusive, or restenotic lesions in the SFA, proximal popliteal artery, or both with lesion length being more than 10 cm; and had at least 1 patent vessel of infrapopliteal runoff to the foot with less than 50% stenosis. Concomitant interventions for iliac and tibial lesions were allowed. Patients with life expectancies of less than 12 months, target lesion within or adjacent to an aneurysm, angiographic evidence of intra-arterial thrombus with contraindications for aspirin or clopidogrel, overwhelming life-threatening infection, in-stent restenosis, the use of drug-eluting devices, a follow-up duration of less than 6 months in patients who are still alive or nonamputated, or procedure refusal were ineligible for inclusion in this study.

A total of 29 patients with 32 legs received ELA with spot stent (group A) while another 76 patients with 87 legs underwent primary stenting (group B). Preinterventional study comprised clinical examination, hemodynamic evaluation (ankle or toe pressure, pulse volume recording (PVR), and duplex ultrasound), and anatomic assessment, including computed tomographic angiography, magnetic resonance angiography, or diagnostic angiography. Toe pressures, PVR, and Doppler waveform patterns were obtained to measure the hemodynamic changes in patients with falsely elevated ankle brachial index (ABI) values. Antecubital venous samples were also obtained before EVI and at 48 hours after EVI to determine C-reactive protein (CRP) levels. A high-sensitivity assay was used to measure serum CRP values. Demographic and interventional data, including clinical presentation according to the Rutherford classification, lesion anatomy based on the Trans-Atlantic Intersociety Consensus (TASC) II system, and the follow-up ABIs, toe pressures, and duplex ultrasounds were recorded for each patient.

The protocol was approved by the local ethics committee and the institutional review board and all study procedures were conducted in accordance with good clinical practice and the applicable laws of various governing bodies.

### 2.1. Endovascular Procedure

The interventional procedure was usually conducted using either the antegrade or crossover approach and also through multiple access sites (distal SFA or pedal puncture) for complex cases. All patients received 100 mg aspirin and 300 mg clopidogrel before the EVI. Unfractionated heparin (5,000–10,000 units) was administered during the procedure to maintain an activated coagulation time around of 250 seconds. The preferred technique in group A was intraluminal recanalization, while avoiding the intentional subintimal passage. If the guidewire failed to cross the obstruction, a step-by-step technique was conducted in which upfront laser ablation was sequentially followed by guidewire advancement. ELA was performed using the Spectranetics CVX-300 pulsed excimer (XeCl) laser system, working at a wavelength of 308 nm (pulse width: 120 ns, fluence: 45–80 mJ/mm^2^, and pulse repetition rate: 25–80 Hz) after wire crossing the stenoocclusive lesions. Laser catheters between 0.9 and 2.5 mm in size were used. In two cases, the Turbo-Booster directional catheter was used to achieve more debulking of the lesions. Balloon PTA was used to optimize the reference vessel diameter after laser ablation. Stents were implanted only for cases with flow-limiting dissection or suboptimal results in group A.

Self-expanding nitinol stents were implanted in group B either by direct stenting or with predilatation of undersized balloon. One centimeter of overlap was employed when multiple stents were required to cover the treated arterial segments. Various types of self-expanding nitinol stents (Protégé/EverFlex (3), ev3 Plymouth, MN, USA; Zilver and Zilver Flex (25), Cook, Bjaeverskov, Denmark; Limerick, Ireland; LifeStent (86), Bard, Peripheral Vascular, Tempe, AZ, USA; Xpert (15), Abbott Vascular, Redwood City, CA, USA; and Marius (36), Invatec, Roncadelle, Italy) were used during the EVI at the discretion of the operators. Aspirin and cilostazol were administered continuously after the EVI if no contraindication was noted. Clopidogrel was used for 3 months after stent implantation.

### 2.2. Angiographic Evaluation

Angiograms were acquired in at least 2 orthogonal views at baseline and after the intervention. A radiopaque ruler was used for the calibration of angiographic measurements, including the length and minimum lumen diameter (MLD) of the target lesion and the mean proximal and distal reference vessel diameter (RVD). The percent diameter stenosis (%DS) was calculated [%DS = (1 − MLD/RVD) × 100] at baseline and after the intervention. In addition, the distal runoff vessels were assessed upon the completion of angiograms to detect the evidence of distal embolization.

### 2.3. Endpoints

The primary endpoint of this study was binary restenosis rate and primary patency rate (PP) at 12 and 24 months. Binary restenosis was defined as a 50% lumen diameter reduction as shown by conventional or digital subtraction angiography during the follow-up or a >50% hemodynamic stenosis determined by duplex ultrasound based on a ≥2.5 peak systolic velocity ratio. The secondary endpoints included technical success, stent fracture, major cardiovascular clinical events (MACE), target lesion revascularization (TLR), assisted primary patency (APP), and amputation-free survival (AFS) rates. Detailed definition of each outcome is as follows.

Technical success was defined as <30% residual stenosis of target lesion after EVI and at least 1 patent tibioperoneal vessel to the distal pedal arch.

Stent fracture rate, determined at the 12- and 24-month follow-ups through X-ray imaging, was categorized as mild (fracture of 1 strut), moderate (fracture ≥ 1 strut but without complete separation), or severe (complete separation) [8].

Target lesion revascularization was defined as any repeat percutaneous intervention of the target lesion because of clinical recurrence of ischemic symptoms and a decrease in ABI of >0.2 coupled with restenosis detected by duplex ultrasound surveillance.

Primary patency was defined as persistent patency without recurrent symptoms in the face of worsening ABIs and a dampened Doppler waveform pattern due to recurrent disease.

Assisted primary patency was defined as the patency achieved after the reintervention for restenosis or reocclusion of the treated vessel.

Limb salvage was defined as freedom from above-ankle amputation of the index limb. Major cardiovascular clinical events rate at 30 days included all-cause mortality, myocardial infarction, stroke, unplanned target limb amputation, procedure-related serious adverse events, device failure, and TLR.

### 2.4. Patient Follow-Up

After being discharged from the hospital, all patients were followed up at an outpatient clinic. Clinical examination and duplex ultrasounds were performed 1 week, 1 month, and 3 months after the index procedures and every 3 months thereafter. Stent fractures were assessed by biplane X-rays at 12 and 24 months in 2 oblique views under the highest magnification with the leg extended and the knee bent. The intervention was repeated if recurrent symptoms, significant vessel stenosis (≥70%) with dampened Doppler waveform patterns shown by the duplex ultrasound, and an ABI decrease of ≥0.2 were observed. Major events (mortality, limb amputation, lesion restenosis, and repeat revascularization) were documented at the time of hospital discharge or during the 3-month follow-up office visits. If office follow-ups were not feasible, telephone interviews, medical records, local electronic medical databases, and referring physicians were used as alternate data sources.

### 2.5. Statistical Analysis

Categorical variables were reported as counts and percentages, and continuous variables were reported as the mean ± standard deviation. Continuous variables were analyzed using *t*-tests whereas Fischer-exact or *χ* ^2^ tests were used for categorical variables. Rates of PP, APP, and AFS in both groups were assessed using the Kaplan-Meier analysis. For the survival analyses, a census of the surviving patients was conducted based on data collected on the day of the last clinical contact. All analyses were performed using Stata 10 (StataCorp, College Station, TX). Statistical significance was set at *P* < 0.05.

## 3. Results

### 3.1. Baseline Patient and Lesion Characteristics

Baseline demographics and clinical characteristics of both treatment groups are summarized in Table 1. Baseline demographics (gender and age) and preexisting medical illness (diabetes mellitus, hypertension, coronary or cerebral artery disease, congestive heart failure, smoking status, or hyperlipidemia) had no differences between the 2 groups. The preprocedural ABIs and the baseline CRP level were also similar between the 2 groups.

A total of 119 femoropopliteal arterial segments were treated in this study population, with 32 in group A and 87 in group B. Lesion classifications according to TASC II, severities of vessel calcification, and numbers of multilevel intervention of the 2 treatment groups were similar (Table 2). The MLDs, RVDs, and %DS before treatment were not different between the 2 groups. The mean lesion lengths of the 2 groups were also similar (16.1 ± 7.6 cm versus 16.2 ± 7.6 cm, *P* = 1.0).

### 3.2. Immediate Procedural Results (Table 3)

The technical success rates were 100% in the 2 treatment groups. In the immediate angiographic results, group B had a significantly larger MLD (4.08 ± 0.80 versus 4.58 ± 0.57 mm, *P* = 0.0002), longer stent length (12.25 ± 6.83 cm versus 17.34 ± 7.76 cm, *P* = 0.0014), more stent implantations (0.75 ± 0.68 versus 1.60 ± 0.73, *P* < 0.0001), and more stent overlappings (13% versus 49%, *P* < 0.001) when compared to group A. Significant reduction of major and minor dissection rates occurred in group A (6% versus 46%, *P* < 0.001 and 13% versus 31%, *P* = 0.031, resp.). In addition, greater increase in CRP level was noted in group A compared to group B (2.98 ± 0.97 versus 0.60 ± 0.72 mg/dL, *P* < 0.001) after the EVI. One vessel perforation occurred during laser angioplasty for popliteal occlusion and was successfully corrected by stent-graft implantation. Distal embolizations occurred twice in group A and once in group B and were successfully treated by catheter aspiration. The ABIs after intervention had no difference between the 2 groups.

### 3.3. In-Hospital Outcomes (Table 4)

There is no procedure-related death or myocardial infarction in the 2 groups. Two patients in group B developed ischemic stroke after the procedure and were left with the sequela of mild hemiparesis. Groin complications occurred in 3 patients in group A and 2 patients in group B but there was no statistical significance. The rates of in-hospital MACE were not different between the 2 groups (9% versus 8%, *P* = 0.51).

### 3.4. Postprocedure Follow-Up (Table 5)

**Figure 1:**
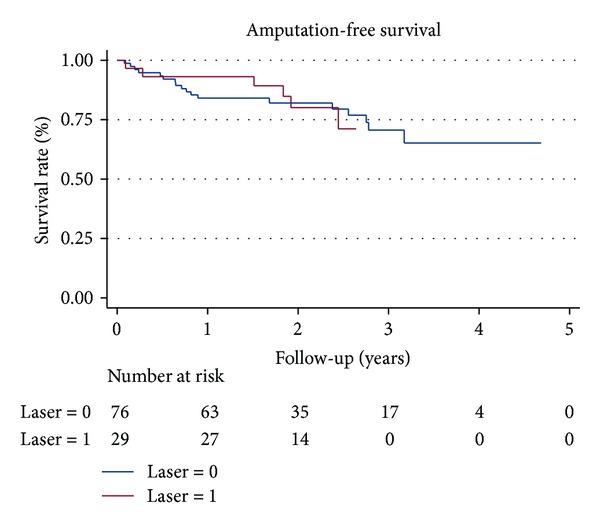
The Kaplan-Meier curve for amputation-free survival. Laser 1 and laser 0 mean group A and group B, respectively.

**Figure 2:**
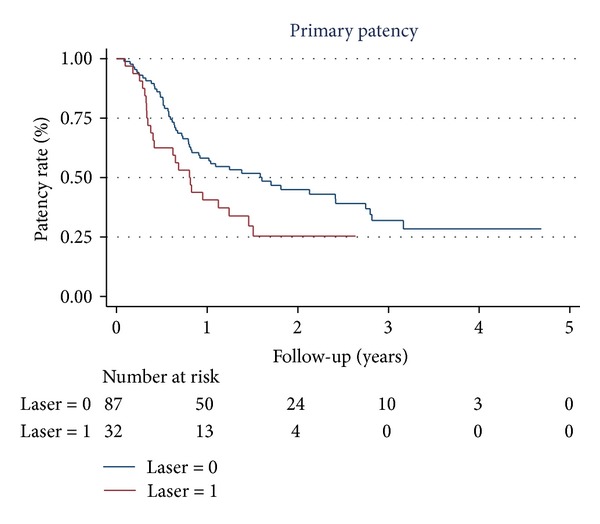
The Kaplan-Meier curve for primary patency. Laser 1 and laser 0 mean group A and group B, respectively.

**Figure 3:**
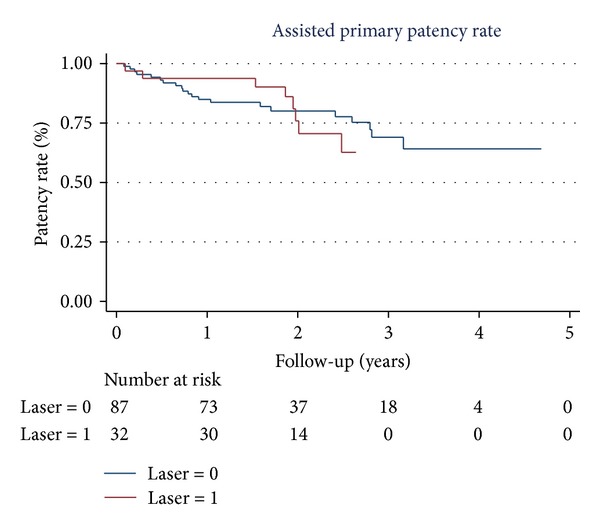
The Kaplan-Meier curve for assisted primary patency. Laser 1 and laser 0 mean group A and group B, respectively.

Eighty-nine percent of study patients were followed up for more than 12 months (93% in group A and 87% in group B) and 2 patients in group B were lost to follow-up 1 year after index procedure. There was no difference in the mean follow-up time between the 2 groups (710 ± 242 versus 766 ± 426 days, *P* = 0.48). Nineteen patients died (5 in group A and 14 in group B) and 6 patients (1 in group A and 5 in group B) underwent major amputations during the follow-up period. The AFS rate was not different in the 2 groups at 24 months (80% versus 82%, *P* = 0.979) (Figure 1). Group A had a significantly higher rate of binary restenosis when compared to group B at 6, 12, and 24 months (37% versus 14%, *P* = 0.007; 67% versus 32%, *P* = 0.001; 83% versus 56%, *P* = 0.028, resp.). Thus, the PP rate was significantly different at 12 and 24 months (40% versus 58%, 25% versus 45%, *P* = 0.039) in the 2 groups (Figure 2). Although the primary stenting group showed more favorable results in the rate of TLR at 6 and 12 months (30% versus 9%, *P* = 0.005 and 53% versus 32%, *P* = 0.018, resp.), this benefit was lost at 2 years. Similar results in the TLR (65% versus 45%, *P* = 0.11) and the APP rates (78% versus 80%, *P* = 0.75) (Figure 3) were observed in the 2 groups at 24 months. The rates of stent fracture were also not different between the 2 groups either at 12 or 24 months (0 versus 2.5%, *P* = 0.51 and 6.3% versus 6.8%, *P* = 0.71, resp.).

## 4. Discussion

This study demonstrated that greater vascular inflammation after ELA with spot stent resulted in earlier restenosis and inferior 1-year clinical outcomes than primary stenting in the treatment of intermediate to long femoropopliteal lesions inspite of the reduced need for stent implantation. Late catch-up restenosis in primary stenting group leads to loss of benefit with similar TLR and APP rates at 2 years between the 2 groups.

The excimer laser application has been studied in long SFA occlusions, which are sometimes made up of several focal stenoses that appear angiographically as a lengthy total occlusion. Debulking the segment with the excimer laser can uncover these more modest lesions that can then be focally treated with balloon angioplasty and, if necessary, spot stenting [10–12]. Scheinert et al. analyzed 318 patients who underwent ELA of 411 lesions averaging 19.4 ± 6.0 cm in length with a 90.5% procedure success rate. Stents were placed only in 30 (7.3%) of the limbs. The primary patency rate was 33%, but the 1-year assisted primary and secondary patency rates were 65.1% and 75.9% under aggressive surveillance monitoring [11]. In the present study, the PP rate of ELA with spot stenting at 12 months was 40% and the binary restenosis rate in group A was significantly higher than that in group B during the follow-up period. Interestingly, all earlier restenoses in group A occurred in the stent-sparing segment. The lower patency in group A might be related to inadequate debulking by Turbo Elite laser catheter, smaller MLD from vessel recoil after the intervention, and, most importantly, excess smooth muscle hyperplasia owing to laser-related vascular inflammation.

C-reactive protein (CRP) is a novel biomarker of vascular inflammation. Intimal and medial injury after balloon angioplasty of coronary and peripheral arteries induces the perivascular inflammatory response [13]; significantly higher postinterventional increase of CRP values was reported in patients undergoing PTA of femoropopliteal artery as compared to those of patients who underwent low extremity angiography [14]. Schillinger et al. reported 172 patients undergoing EVI for femoropopliteal lesions and showed that greater increase in CRP levels after revascularization was associated with higher restenosis rates and lower ABI levels at the 6-month follow-up [15]. In the present study, significant increase in CRP levels was observed in group A after EVI, which represented higher perivascular inflammatory response, perhaps due to more intimal and medial injuries by laser photoablation than mechanical stretch by long stent implantation. Our observation suggested that the extent of post-EVI inflammation at treated vessel segments contributes significantly to more neointimal hyperplasia and earlier restenosis. Although the CELLO study has used the Turbo-Booster laser guide catheter to increase lumen diameter and maximize plaque removal, the PP rate was 59% at 6 months and 54% at 1 year. TLR was required in 23.1% of CELLO participants at 1 year [16]. However, the mean lesion length in this study was relatively short (5.6 ± 4.7 cm). It is unclear whether these results can be applied to longer femoropopliteal lesions.

Nitinol stents were introduced several years ago and have demonstrated superior primary patency to balloon angioplasty [3, 7]. Data from a meta-analysis has demonstrated that the 12-month binary restenosis rate was significantly lower in the primary stenting group compared with that in the balloon angioplasty group (OR 3.02, 95% CI 1.3–6.71, *P* < 0.001) [17]. However, the mean lesion length of these trials was 7.46 cm and the promising result cannot be applied to longer lesions.

There is a paucity of data in the literature regarding the long-term outcomes of stenting in intermediate to long femoropopliteal lesions. Thus, patients with lesion lengths more than 10 cm were enrolled in this study and 64% of group B patients had TASC II C and D lesions. The primary patency rates at 1 year and 2 year were 58% and 45%, which were inferior to those reported by Iida et al. [18]. However, more TASC II C and D patients were included in this study. Previous single-institution studies have also reported low primary patency rates (27.5–36% at 2 years) of primary stenting in TASC II C and D lesions [19, 20].

We have reported the comparison of the 1-year outcomes between these 2 groups previously [21]. For longer follow-up time, the late catch-up restenosis in group B resulted in no difference in TLR between the 2 groups at 2 years. Iida et al. reported that the peak timing of restenosis following nitinol stent in SFA was the 369th day and that 1-year observation might be too short [22]. In the present study, most patients were followed for more than 12 months. Chronic inflammatory reaction between nitinol stent and endothelium persisted even after 1 year, which led to late neointima hyperplasia and restenosis in the primary stenting group.

Scheinert et al. have reported a 37.2% stent fracture rate in long-segmental SFA stenting [8]. With the new generation of stent design, the fracture rate was reduced to 3.1–6% [3, 4]. In this study, there was no difference in the stent fracture rates between spot stenting and long stenting during the 2-year follow-up, perhaps because all femoropopliteal lesions in this study were treated with the new generation of stents. Besides, most of the study's patients had critical limb ischemia and the lack of strenuous exercise lessened the risk of stent fracture.

At the end of the study period, the drug-eluting stent (DES) (Zilver PTX, Cook, and Limerick, Ireland) had just been introduced into the country and cases of longer femoropopliteal lesions treated with DES were limited. Bosiers et al. reported the single arm trial of DES in the treatment of long femoropopliteal lesions (mean length of 226.1 ± 43.6 mm) with rates of 77.6% of PP, 85.4% of freedom from TLR, and 2.1% of stent fracture at 12 months [23]. A meta-analysis also showed the superiority of drug eluting balloon over uncoated balloon in reducing the TLR of femoropopliteal intervention [24]. Drug-eluting devices might attenuate the vascular inflammation, hold promises to improve the vessel patency, and reduce the need of metallic implant in the treatment of intermediate to long femoropopliteal lesions.

### 4.1. Study Limitations

This study is a retrospective analysis of a prospectively maintained database but the relatively small sample size limits the statistical significance of the results. Single-institution series are often biased toward particular patient demographics and practice pattern, but these data represent the real-world application of ELA in low extremity revascularization. No wide use of Turbo-Booster catheter in this study might have caused inadequate debulking in the ELA with spot stent group. Routine follow-up angiography was not conducted, and thus the quantitative measurement of late lumen loss was not available. The lack of CRP levels at follow-up also resulted in difficulty in the comparison of chronic vascular inflammation between the 2 groups.

In conclusion, for patients with intermediate to long femoropopliteal disease, earlier restenosis and inferior 1-year clinical outcomes were found in ELA with spot stent due to greater perivascular inflammation when compared to primary stenting group. However, this benefit in primary stenting group was lost at 2 years due to late catch-up restenosis. Active surveillance with prompt intervention is required to maintain the vessel patency.

## Figures and Tables

**Table 1:** Baseline demographics.

	Gr A		Gr B		*P* value
Patient No.	29		76		
Age	71 ± 11		74 ± 11		0.21
Gender: male	16	(55%)	35	(46%)	0.37
Underlying disease					
Diabetes Mellitus	24	(83%)	65	(86%)	0.72
Hypertension	23	(79%)	66	(86%)	0.34
CAD/CVA	22	(75%)	52	(69%)	0.45
Chronic renal failure or dialysis dependence	20	(69%)	47	(62%)	0.49
CHF	3	(10%)	8	(11%)	1.0
Smoking	9	(31%)	22	(32%)	0.83
Hyperlipidemia	18	(62%)	46	(61%)	0.89
Treated limbs	*N* = 32		*N* = 87		
Target limb ABI	0.46 ± 0.13		0.47 ± 0.16		0.75
CRP (mg/dL)	1.71 ± 2.14		2.54 ± 3.79		0.27
Clinical presentation					
Intermittent claudication	12	(37%)	18	(21%)	0.06
Rest pain	8	(25%)	16	(18%)	0.43
Unhealing ulcer	8	(25%)	37	(43%)	0.08
Gangrene	4	(13%)	16	(18%)	0.45

ABI: ankle brachial pressure index; CAD: coronary artery disease; CRP: C-reactive protein; CVA: cerebrovascular accident; CHF: congestive heart failure.

**Table 2:** Baseline lesion characteristics of femoropopliteal artery.

	Gr A (*N* = 32)		Gr B (*N* = 87)		*P* value
Concomitant intervention					
Iliac intervention	2	(6%)	5	(6%)	1.00
Tibial intervention	18	(56%)	50	(57%)	0.91
TASC II classification					
B	9	(28%)	31	(36%)	0.44
C/D	23	(72%)	56	(64%)	
Lesion classification					
De novo stenosis	26	(81%)	80	(92%)	0.097
Restenosis	6	(19%)	7	(8%)	
Occlusion	18	(56%)	37	(43%)	0.18
Lesion calcification					
Mild	3	(9%)	9	(10%)	0.95
Moderate	12	(38%)	36	(41%)	
Severe	17	(53%)	42	(49%)	
RVD	4.99 ± 0.67 mm		4.94 ± 0.67 mm		0.72
MLD	0.46 ± 0.54 mm		0.65 ± 0.68 mm		0.16
Degree of stenosis (%)	90 ± 18		87 ± 14		0.34
Mean lesion length	16.1 ± 7.6 cm		16.2 ± 7.6 cm		0.94

MLD: minimal lumen diameter; RVD: reference vessel diameter; TASC: trans-atlantic intersociety consensus.

**Table 3:** Immediate procedural results.

	Gr A (*N* = 32)		Gr B (*N* = 87)		*P* value
Postprocedural results					
Reference vessel diameter	5.02 ± 0.68 mm		5.15 ± 0.56 mm		0.29
Minimal lumen diameter	4.08 ± 0.80 mm		4.58 ± 0.57 mm		0.0002
Degree of stenosis (%)	18 ± 10		11 ± 6		0.0001
Adjuvant stent implantation					
Mean stent length	12.25 ± 6.83 cm		17.34 ± 7.76 cm		0.0014
Mean stent numbers per leg	0.75 ± 0.68 (24/32)		1.60 ± 0.73 (139/87)		<0.0001
Stent type					
Single stent	16/32	(50%)	44/87	(51%)	<0.0001
Overlapping stent	4/32	(13%)	43/87	(49%)	
Without stenting	12/32	(37%)	0	(0%)	
Complications					
Perforation	1		0		0.27
Distal embolization	2	(6%)	1	(1%)	0.18
Major dissection	2	(6%)	40	(46%)	<0.001
Minor dissection	4	(13%)	27	(31%)	0.031
ABI after EVI	0.92 ± 0.09		0.92 ± 0.15		1.00
CRP after EVI (mg/dL)	4.69 ± 2.41		3.14 ± 4.15		0.049
Change of CRP levels (mg/dL)	2.98 ± 0.97		0.60 ± 0.72		<0.001

ABI: ankle brachial pressure index; CRP: C-reactive protein; EVI: endovascular intervention.

**Table 4:** In-hospital and 30-day outcomes.

	Gr A	Gr B	*P* value
Death	0	0	
MI	0	0	
CVA	0	2	
Emergent surgery	0	0	
Groin complications	3	2	
UGI bleeding with shock	0	2	
Compartment syndrome	0	1	
MACE	3/32 (9%)	7/87 (8%)	*P* = 0.51

CVA: cerebrovascular accident; MI: myocardial infarction; MACE: major cardiovascular clinical events; UGI: upper gastrointestinal tract.

**Table 5:** Follow-up results.

	Gr A (*N* = 32)	Gr B (*N* = 87)	*P* value
Mean follow-up	710 ± 242	766 ± 426 days	0.48
Binary restenosis rate (by duplex US PSV ratio > 2.5)			
6 months	11/30 = 37%	11/80 = 14%	0.007
12 months	20/30 = 67%	24/74 = 32%	0.001
24 months	19/23 = 83%	31/55 = 56%	0.028
Target vessel revascularization rate			
6 months	9/30 = 30%	7/80 = 9%	0.005
12 months	16/30 = 53%	21/73 = 29%	0.018
24 months	15/23 = 65%	24/53 = 45%	0.11
Stent fracture rate			
12 months	0	3/122 (2.5%)	0.51
24 months	1/16 (6.3%)	5/74 (6.8%)	0.71
